# Utility of the sore throat pain model in a multiple-dose assessment of the acute analgesic flurbiprofen: a randomized controlled study

**DOI:** 10.1186/1745-6215-15-263

**Published:** 2014-07-03

**Authors:** Bernard Schachtel, Sue Aspley, Adrian Shephard, Timothy Shea, Gary Smith, Emily Schachtel

**Affiliations:** 1 Department of Epidemiology & Public Health, Yale University School of Medicine, 60 College Street, New Haven, CT 06520-8034, USA; 2 Schachtel Research Company, Inc, 4300 So. US Highway One, Suite 203, Jupiter, FL 33477, USA; 3 Reckitt Benckiser Healthcare International Ltd., 103-105 Bath Road, Slough, Berkshire SL1 3UH, UK

**Keywords:** Acute pain, Flurbiprofen, Nonsteroidal anti-inflammatory agent, Pharyngitis, Sore throat

## Abstract

**Background:**

The sore throat pain model has been conducted by different clinical investigators to demonstrate the efficacy of acute analgesic drugs in single-dose randomized clinical trials. The model used here was designed to study the multiple-dose safety and efficacy of lozenges containing flurbiprofen at 8.75 mg.

**Methods:**

Adults (n = 198) with moderate or severe acute sore throat and findings of pharyngitis on a Tonsillo-Pharyngitis Assessment (TPA) were randomly assigned to use either flurbiprofen 8.75 mg lozenges (n = 101) or matching placebo lozenges (n = 97) under double-blind conditions. Patients sucked one lozenge every three to six hours as needed, up to five lozenges per day, and rated symptoms on 100-mm scales: the Sore Throat Pain Intensity Scale (STPIS), the Difficulty Swallowing Scale (DSS), and the Swollen Throat Scale (SwoTS).

**Results:**

Reductions in pain (lasting for three hours) and in difficulty swallowing and throat swelling (for four hours) were observed after a single dose of the flurbiprofen 8.75 mg lozenge (*P* <0.05 compared with placebo). After using multiple doses over 24 hours, flurbiprofen-treated patients experienced a 59% greater reduction in throat pain, 45% less difficulty swallowing, and 44% less throat swelling than placebo-treated patients (all *P* <0.01). There were no serious adverse events.

**Conclusions:**

Utilizing the sore throat pain model with multiple doses over 24 hours, flurbiprofen 8.75 mg lozenges were shown to be an effective, well-tolerated treatment for sore throat pain. Other pharmacologic actions (reduced difficulty swallowing and reduced throat swelling) and overall patient satisfaction from the flurbiprofen lozenges were also demonstrated in this multiple-dose implementation of the sore throat pain model.

**Trial registration:**

This trial was registered with ClinicalTrials.gov, registration number: NCT01048866, registration date: January 13, 2010.

## Background

The sore throat pain model has been used to demonstrate the efficacy of acute analgesic drugs and dosages of these drugs in single-dose randomized placebo-controlled studies [1-4]. As for most acute pain conditions, patients with painful pharyngitis due to upper respiratory tract infection (URTI) often require repeated treatment beyond one or two doses, especially on the first day of treatment, until the URTI resolves [5,6]. However, the *vis medicatrix naturae* of sore throat [7-9], earache [10], and other types of acute pain [11,12] makes it difficult to distinguish an active analgesic from placebo when treatments are re-tested over time, even over 24 hours.

Recognizing this investigational challenge in examining multiple-dose efficacy in patients with an acute, self-limited infectious disease, we employed four specific principles of research architecture in order to tighten the design of this multiple-dose trial. First, to avoid confusion with other expressions of URTI that can also cause a sore throat (such as laryngitis) and might respond differently to treatment, we enrolled only patients with sore throat due to pharyngitis. Homogeneity of diagnosis was confirmed on the Tonsillo-Pharyngitis Assessment (TPA), an index of distinct clinical features of pharyngitis [1,13,14]. Measurements on the TPA also helped confirm that patients in the treatment groups had the same severity of the physical condition causing sore throat.

Second, to fully characterize the many expressions of throat *dis*-ease which the patients described, we sought to evaluate other dimensions of throat pain in addition to the evaluative quality of ‘sore’ throat (pain intensity). We asked the patients to evaluate two other prominent throat-related symptoms which they also complained of, the sensation of a ‘swollen throat’, a sensory quality commonly reported by patients with sore throat, as well as ‘difficulty swallowing’, a throat function (dysphagia, distinctly different from odynophagia) [4,5,15-18].

Third, because patients with acute sore throat require treatment mostly during the early course of disease, we sought to enroll patients within three days of their first throat symptom (even a mild sore throat, such as a ‘throat tickle’). Thus, we would be more likely to observe patients who provided assessments of sore throat throughout the first 24 hours of the clinical trial. Also, to help assure that symptom severity would be more likely to persist for at least 24 hours and efficacy could be assessed over this treatment period, patients were eligible only if they reported at least a moderate severity of sore throat pain, swollen throat, and difficulty swallowing at baseline.

Previous studies have demonstrated the efficacy and safety of a single dose of a lozenge containing the nonsteroidal anti-inflammatory drug flurbiprofen in patients with sore throat [7-9,19,20], including a single-dose study on different dosages of flurbiprofen lozenge [21]. Here we present the results of a clinical trial utilizing the sore throat pain model to examine the efficacy and safety of multiple doses of a lozenge containing flurbiprofen 8.75 mg, with a focus on the initial 24 hours of treatment.

## Methods

### Study design

This was a randomized (ratio 1:1), double-blind, placebo-controlled, multiple-dose, parallel-group study. The first subject was enrolled in November 2009 and the last subject completed in March 2011. The study was conducted in accordance with the International Conference on Harmonization Good Clinical Practice and the ethical principles contained within the Declaration of Helsinki (South Africa, 1996). The study also complied with the Code of Federal Regulations (CFR) of the United States Food and Drug Administration (FDA) and the United States Good Clinical Practice Regulations. The study protocol was reviewed and approved by The New York University School of Medicine’s Institutional Review Board, Compass Institutional Review Board and IntegReview Ethical Review Board. The study was registered with the ClinicalTrials.gov registry, registration number: NCT01048866.

### Patient selection

**Figure 1:**
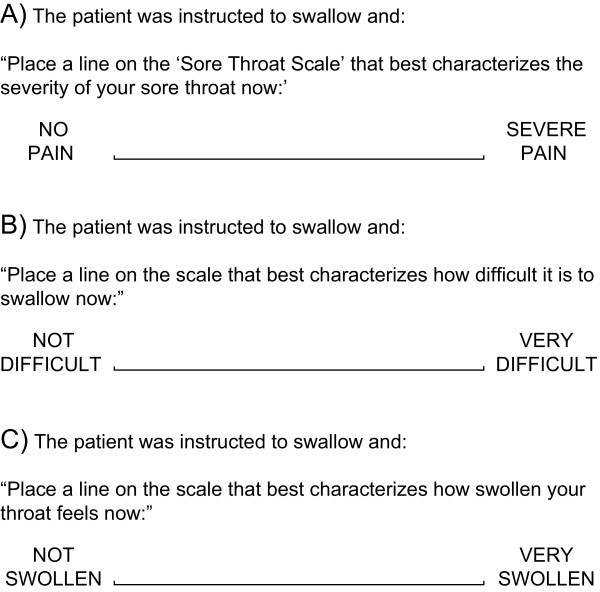
**Visual analog scales to assess patient-reported outcomes.** The 100-mm visual analog scales used were **A)** the Sore Throat Pain Intensity Scale (STPIS) to assess sore throat pain, **B)** the Difficulty Swallowing Scale (DSS) to assess difficulty swallowing, and **C)** the Swollen Throat Scale (SwoTS) to assess the sensation of a swollen throat.

Patients were screened by the study investigators at research sites in the United States after seeking medical care for sore throat, being referred to the trial site, or responding to advertisements about this study. All participants provided written informed consent prior to participation in the study. Adults (≥18 years) were included if they had a sore throat of recent onset (≤3 days), moderate or severe pain on the Throat Pain Scale (a four-category pain intensity scale), at least one symptom of URTI on the URTI Questionnaire [2,14], objective findings of pharyngeal inflammation (≥5 on the TPA; Table 1) [1,13,14], and throat symptoms on three patient-reported outcome measures: sore throat pain rated more than or equal to 66 mm on the Sore Throat Pain Intensity Scale (STPIS); difficulty swallowing rated more than or equal to 50 mm on the Difficulty Swallowing Scale (DSS); and the sensation of a swollen throat rated more than or equal to 33 mm on the Swollen Throat Scale (SwoTS). See Figure 1 for descriptions of the three symptom rating scales.

**Table 1:** Tonsillo-Pharyngitis Assessment (TPA)

**Finding**	**0 Points**	**1 Point**	**2 Points**	**3 Points**
Oral temperature	≤ 98.6°F	98.7 – 98.9°F	99.0 – 99.9°F	≥ 100.0°F
Oropharyngeal color	Normal/pink	Slightly red	Red	Beefy red
Size of tonsils	Normal/absent	Slightly enlarged	Moderately enlarged	Much enlarged
Number of oropharyngeal enanthems (vesicles, petechiae, or exudates)	None	Few	Several	Many
Largest size of anterior cervical lymph nodes	Normal	Slightly enlarged	Moderately enlarged	Much enlarged
Number of anterior cervical lymph nodes	Normal	Slightly increased	Moderately increased	Greatly increased
Maximum tenderness of some anterior cervical lymph nodes	Not tender	Slightly tender	Moderately tender	Very tender

Patients were ineligible if they displayed confounding features of URTI: mouth breathing which causes throat drying, coughing which causes throat discomfort, or any disease that could compromise respiratory function. Patients were also ineligible if they had a history of allergic reaction or hypersensitivity to aspirin or other nonsteroidal anti-inflammatory drugs, or any gastrointestinal, hepatic, or renal dysfunction. Patients were excluded if they had used any throat lozenge, throat spray, cough drop, or menthol-containing product within one hour prior to study screening, used any ‘cold medication’ or immediate-release analgesic within the previous four hours, used any sustained-release analgesic within the previous 12 hours, taken an antibiotic for an acute disease within 24 hours, or any quinolone antibiotic or inhaled therapy in the previous week.

### Study medications

Under double-blind conditions, patients were randomly allocated to receive sugar-based, flavored flurbiprofen 8.75 mg lozenges or sugar-based, identically flavored placebo lozenges (containing the same vehicle ingredients as the flurbiprofen lozenge). Randomization was achieved using a computer-generated randomization schedule provided by a statistician who was not involved in the analysis of the study. The patients, study investigators, and all other study personnel remained blinded to the treatment allocation after randomization.

Patients were instructed to suck one lozenge (flurbiprofen 8.75 mg or placebo) and were then not permitted to have anything by mouth for the next two hours. Patients were assessed onsite for the first two hours, then discharged with a supply of the same trial lozenges (one lozenge to be used as needed every three to six hours, up to five lozenges in a 24-hour period) and acetaminophen 650 mg tablets (to be taken as needed every four to six hours if there was inadequate relief from the trial lozenge). No other medications were permitted during this 24-hour period. Over 24 hours (while awake), patients used a diary to record hourly assessments of sore throat pain, swollen throat, and difficulty swallowing, their medication consumption, and any adverse events (AEs). A follow-up visit was conducted for the 24-hour assessments. Over the following six days patients assessed secondary outcomes before and after each self-dosing. At the end of the seven-day observation period patients returned to the site for final assessments and review of AEs and were discharged from the study.

### Assessments

At baseline, all patients were examined for physical findings of tonsillo-pharyngitis, as measured on the TPA (Table 1), and for a global assessment of the severity of pharyngeal inflammation, on the categorical Practitioner’s Assessment of Inflammation, or PrAoI (none, mild, moderate, severe). All patients had a throat culture performed to determine the presence of group A beta-hemolytic streptococcus in the oropharynx. Upon receipt of culture results at 24 to 48 hours, all patients with group A infection received antibiotics and symptomatic patients with group C streptococcus infection also received antibiotics. All patients continued in the study after the initial 24 hours and were included in the efficacy analyses regardless of their streptococcus infection or antibiotic treatment status.

Previously validated 100-mm visual analog scales (Figure 1) were used to record the symptoms of sore throat pain intensity, difficulty swallowing, and the sensation of a swollen throat [1,4,18,22,23]. Patients assessed throat symptoms on the STPIS, DSS, and SwoTS at baseline, one hour, and two hours after the first dose, then every hour for the remainder of the first 24-hour study period (when awake), and again at pretreatment, and one and two hours after each dose taken over days two to seven.

Sore throat relief was also measured at one hour and two hours after the first dose on a validated scale, the Sore Throat Relief Rating Scale (STRRS), a six-category scale ranging from ‘no relief’ to ‘complete relief’ [15,23].

At the 24-hour visit patients were asked to rate their satisfaction or dissatisfaction with the study medication by completing the Patient Satisfaction Scale, a seven-category scale ranging from ‘extremely dissatisfied’ to ‘extremely satisfied’ [24].

### Statistical analyses

The primary endpoint of this study was the time-weighted summed difference in pain intensity on the STPIS over 24 hours after the first dose of study medication (SPID24). Based on a previous study utilizing this endpoint [21], and assuming a 20% effect size [7-9] with 80% power, 200 patients were needed for this study (100 patients on each treatment).

Efficacy was calculated using least square (LS) means, and analysis of variance (ANOVA) was used to compare flurbiprofen 8.75 mg and placebo groups, with treatment and site included as a fixed effect and the relevant baseline (baseline STPIS, DSS, or SwoTS score) included as a covariate. The time-weighted summed differences in DSS and SwoTS over 24 hours were similarly evaluated. The time-weighted summed differences for all three outcomes were assessed over two hours after each dosing over days two to seven. If a patient used rescue medication for pain, all subsequent STPIS, DSS, and SwoTS scores in the 24-hour interval were assigned the baseline value (baseline observation carried forward, BOCF). Missing scores for time-weighted summed differences from baseline were imputed using linear interpolation, assuming the time of the missing assessment to be the nominal time since the first dose, in order to give a more reliable approximation of the AUC from the non-missing data. Two-sided statistical tests were performed with significance determined by reference to the 5% significance level.

Wilcoxon rank sum tests were performed to analyze the data obtained for the pharyngeal inflammation category (on the PrAoI), duration of sore throat, size of tonsils (on the TPA), sore throat relief, and patient satisfaction. The chi-square test was performed to analyze the sex and AE data, and two-sided statistical t-tests were used to analyze the other parameters assessed at baseline.

To determine the efficacy of a single dose of study medication as measured on the STPIS, DSS, and SwoTS [1,4,5,22,23], the absolute change from baseline was calculated over the six hours after the initial single dose of study medication. The odds of achieving at least a 20% reduction in symptom severity (on the STPIS, DSS, or SwoTS) with flurbiprofen 8.75 mg compared with placebo was assessed using logistic regression, after adjusting for site effects. Treatment effect was also analyzed by determining the cumulative percentage of responders (patients who reported at least a 20% reduction from baseline in STPIS score) over the initial six hours. These data were used to calculate the absolute risk difference (ARD) between the proportion of responders for the two treatment groups and to calculate the number needed to treat (NNT; 1/ARD). For patients who used additional study or rescue medication in the initial six-hour treatment period, all subsequent changes in STPIS, DSS, and SwoTS scores over six hours were set to zero according to the BOCF convention. All statistical analyses were performed using SAS version 9.2, SAS Institute Inc., Cary, NC, USA.

## Results

### Patient enrollment, disposition, and demographics

**Figure 2:**
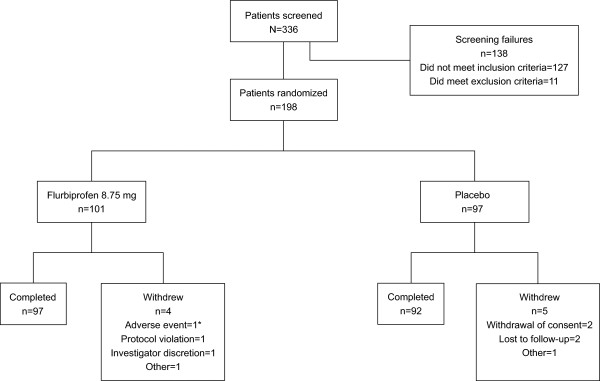
**Patient disposition.** *Discontinuation was due to headache.

Of the 336 patients screened, 198 patients were randomized to receive either flurbiprofen 8.75 mg (n = 101) or placebo (n = 97) lozenges (Figure 2). One patient (in the placebo treatment group) withdrew consent and did not record any information between 2 and 24 hours post initial dose, eight patients (four in each treatment group) discontinued between days two and seven; 189 patients completed the study. Patients in both treatment groups had comparable baseline clinical features (*P* >0.1 for differences between groups). Only 4.1% of the patients had group A beta-hemolytic streptococcal pharyngitis, with no difference between the treatment groups (3.0% in the flurbiprofen 8.75 mg group, 5.2% in the placebo group; *P* >0.1), and most patients had evidence of moderate or severe pharyngeal inflammation (Table 2). The pretreatment severity of all three pharyngeal symptoms (on the STPIS, DSS, and SwoTS) was also comparable (all *P* >0.1). Most patients had moderate pain at baseline (69.3% of patients in the flurbiprofen 8.75 mg treatment group and 66% in the placebo group; *P* >0.1) (Table 2).

**Table 2:** Patient demographics and baseline characteristics

**Characteristic**	**Flurbiprofen 8.75 mg (n = 101)**	**Placebo (n = 97)**	**Overall (n = 198)**
Sex, male, n (%)	40 (39.6)	39 (40.2)	79 (39.9)
*P* value			0.9311
Age (years)			
Mean ± SD	33.5 ± 11.0	34.2 ± 11.2	33.9 ± 11.1
Range	18 – 61	18 – 58	18 – 61
*P* value			0.6477
Duration of sore throat, n (%)			
1 day	24 (23.8)	19 (19.6)	43 (21.7)
2 days	39 (38.6)	31 (32.0)	70 (35.4)
3 days	37 (36.6)	43 (44.3)	80 (40.4)
4 days	0 (0.0)	3 (3.1)	3 (1.5)
5 days	1 (1.0)	1 (1.0)	2 (1.0)
*P* value			0.1250
Pain severity, n (%)			
Moderate	70 (69.3)	64 (66.0)	134 (67.7)
Severe	31 (30.7)	33 (34.0)	64 (32.3)
*P* value			0.6168
Mean TPA score ± SD	8.0 ± 2.26	8.1 ± 2.58	8.1 ± 2.42
*P* value			0.7630
TPA - size of tonsils, n (%)			
Normal or absent	27 (26.7)	25 (25.8)	52 (26.3)
Slightly enlarged	42 (41.6)	37 (38.1)	79 (39.9)
Moderately enlarged	27 (26.7)	31 (32.0)	58 (29.3)
Much enlarged	5 (5.0)	4 (4.1)	9 (4.5)
*P* value			0.6635
PrAoI, n (%)			
No inflammation	0 (0.0)	1 (1.1)	1 (0.5)
Mild inflammation	42 (41.6)	37 (38.5)	79 (40.1)
Moderate inflammation	52 (51.5)	53 (55.2)	105 (53.3)
Severe inflammation	7 (6.9)	5 (5.2)	12 (6.1)
*P* value			0.9550
Mean STPIS score ± SD	79.1 ± 8.1	79.1 ± 8.4	79.1 ± 8.2
*P* value			0.9972
Mean DSS score ± SD	77.9 ± 10.6	78.2 ± 10.4	78.0 ± 10.5
*P* value			0.8173
Mean SwoTS score ± SD	76.0 ± 12.9	76.0 ± 12.8	76.0 ± 12.8
*P* value			0.9739

DSS, Difficulty Swallowing Scale; PrAoI, Practitioner’s Assessment of Inflammation; SD, standard deviation; STPIS, Sore Throat Pain Intensity Scale; SwoTS, Swollen Throat Scale; TPA, Tonsillo-Pharyngitis Assessment.

### Efficacy

**Figure 3:**
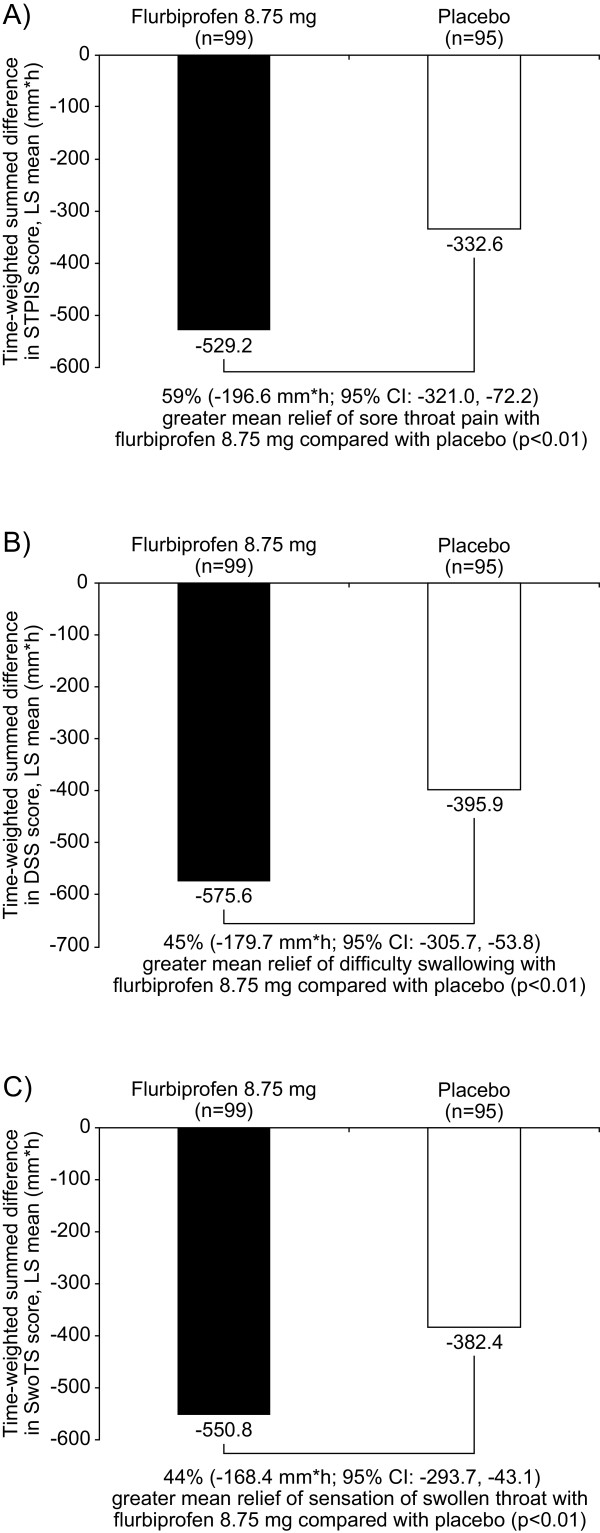
**Efficacy of multiple doses of flurbiprofen 8.75 mg or placebo lozenges over 24 hours.** ANOVA was used to compare flurbiprofen 8.75 mg and placebo groups for the time-weighted summed differences in LS mean scores over 24 hours for **A)** sore throat pain (on the STPIS), **B)** difficulty swallowing (on the DSS), and **C)** swollen throat (on the SwoTS). ANOVA, analysis of variance; CI, confidence interval; DSS, Difficulty Swallowing Scale; h, hour; LS, least square; STPIS, Sore Throat Pain Intensity Scale; SwoTS, Swollen Throat Scale.

On average, patients used 4.7 lozenges (standard deviation (SD) 0.96, range 2 to 8) over the initial 24-hour treatment period. For the study’s primary endpoint (SPID24), patients in the flurbiprofen 8.75 mg group reported a 59% greater reduction in pain than patients taking placebo (difference of –196.6 mm × h; 95% confidence interval (CI) –321.0 to -72.2; *P* <0.01; Figure 3A). Patients taking flurbiprofen 8.75 mg lozenges also experienced a 45% greater relief of difficulty swallowing (difference of -179.7; 95% CI -305.7 to -53.8; *P* <0.01; Figure 3B) and a 44% greater reduction of swollen throat than patients taking placebo lozenges over the 24-hour period (difference of -168.4; 95% CI -293.7 to -43.1; *P* <0.01; Figure 3C). A greater percentage (53.6%) of patients in the flurbiprofen 8.75 mg treatment group reported they were ‘satisfied’, ‘very satisfied’, or ‘extremely satisfied’ with treatment than patients in the placebo group (38.5%; *P* <0.05).

**Figure 4:**
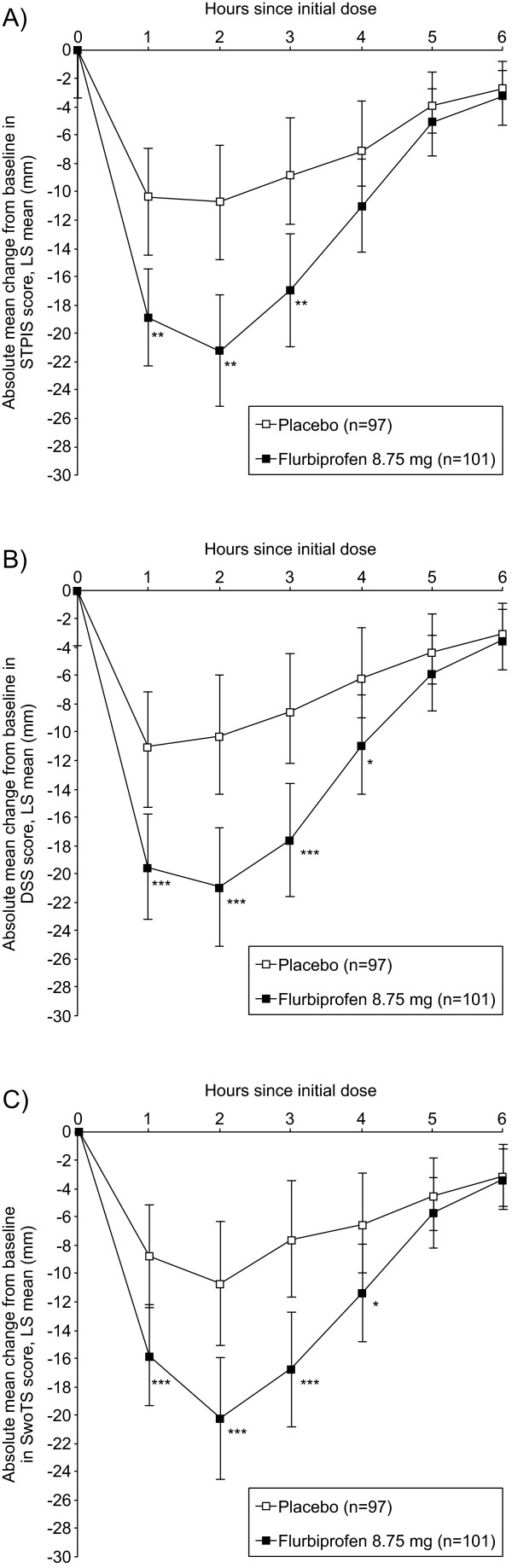
**Efficacy of a single flurbiprofen 8.75 mg or placebo lozenge over six hours.** ANOVA was used to compare flurbiprofen 8.75 mg (n = 101) and placebo (n = 97 up to two hours, n = 96 from three to six hours, as one patient withdrew from the study) for the effects of a single dose on absolute mean change over six hours in **A)** sore throat pain (on the STPIS), **B)** difficulty swallowing (on the DSS), and **C)** swollen throat (on the SwoTS). Error bars represent the 95% CIs. *P <0.05, **P <0.01, ***P <0.001 compared with placebo. ANOVA, analysis of variance; CI, confidence interval; DSS, Difficulty Swallowing Scale; LS, Least square; STPIS, Sore Throat Pain Intensity Scale; SwoTS, Swollen Throat Scale.

**Figure 5:**
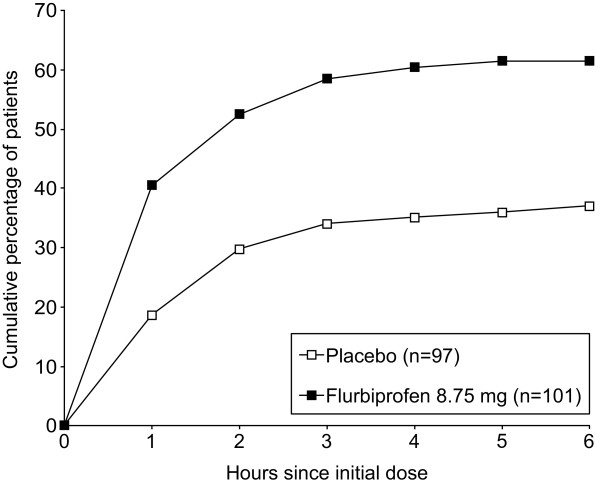
Cumulative percentage of patients achieving at least a 20% reduction from baseline in Sore Throat Pain Intensity Scale (STPIS) score.

When the effects of a single dose were analyzed, a statistically significant reduction in sore throat pain intensity was observed for patients treated with flurbiprofen 8.75 mg lozenge compared with placebo up to three hours post-dose (*P* <0.01). Reductions in absolute DSS and SwoTS levels were also observed at each hourly assessment up to and including four hours post-dose for patients in the flurbiprofen 8.75 mg treatment group compared with placebo (all *P* <0.05) (Figure 4A-C). Significantly greater percentages of flurbiprofen-treated patients reported at least a 20% reduction in sore throat pain intensity than patients in the placebo treatment group (61.4 and 37.1% respectively; *P* <0.001), in difficulty swallowing (57.4 and 36.1% respectively; *P* <0.01), and in swollen throat (63.4 and 40.2%; *P* <0.01). Using a cumulative responder analysis of patients achieving at least 20% reduction in sore throat pain intensity (Figure 5), the ARD between flurbiprofen and placebo was 0.22 (NNT 4.5) at one hour post-dose and 0.24 (NNT 4.1) at six hours post-dose.

Sore throat relief was significantly greater for patients who used the flurbiprofen 8.75 mg lozenge than placebo at one hour (mean (SD) scores 1.8 (1.22) and 1.2 (1.15), respectively; *P* <0.01) and two hours (mean (SD) scores 2.0 (1.23) and 1.3 (1.24); *P* <0.0001), the two time points relief was directly measured (on the STRRS).

Patients taking flurbiprofen 8.75 mg lozenges over days two to seven experienced a 38.0% greater relief of sore throat pain (difference of -8.2; 95% CI -17.1 to 0.7; *P* = 0.07), a 36.9% greater improvement in difficulty swallowing (difference of -7.5; 95% CI -16.2 to 1.3; *P* = 0.09), and a 45.0% greater relief of swollen throat compared with patients taking placebo lozenges (difference of -8.1; 95% CI -16.8 to 0.6; *P* = 0.07).

### Safety

The percentage of patients who reported any AE in the first 24 hours of the study (when most lozenges were consumed) was similar for both treatment groups (25.7% in the flurbiprofen 8.75 mg group, 19.6% in the placebo group; *P* >0.1). Most AEs were related to URTI symptoms (headache and throat irritation). Severe AEs were reported by 1.0% of patients in both treatment groups (*P* >0.1); the incidence of gastrointestinal AEs related to study treatment was also similar between groups (occurring in 3.0 and 1.0% of patients in the flurbiprofen 8.75 mg and placebo treatment groups, respectively; *P* >0.1). Over seven days, the proportion of patients reporting any AE remained similar between the treatment groups (33.7% in the flurbiprofen 8.75 mg group, 28.9% in the placebo group; *P* >0.1), as was the incidence of treatment-related gastrointestinal AEs (3.0% in the flurbiprofen 8.75 mg group, 2.1% in the placebo group; *P* >0.1).

## Discussion

To evaluate the multiple-dose effects of an analgesic-containing lozenge in patients with sore throat, a self-limited inflammatory pain condition, this randomized, double-blind, placebo-controlled study required strict adherence to basic principles of clinical trial design and methodology. By tightening the admission requirements (specificity of diagnosis, acute onset, and severity of throat symptoms), eliminating confounding clinical features, using validated rating scales for different throat symptoms (not only pain intensity), and measuring these symptoms over the first 24-hour period when throat symptoms are most prominent and require repeated treatment, this multiple-dose trial was sufficiently sensitive to differentiate an active analgesic drug from placebo. The patients were able to detect distinct differences between the sugar-based, flavored flurbiprofen 8.75 mg lozenges and the sugar-based, identically flavored placebo lozenges for the five patient-reported outcomes (reduction of sore throat pain, difficulty swallowing, swollen throat, sore throat relief, and overall patient satisfaction).

The study was also sensitive to single-dose effects, confirming results of previous studies on a single flurbiprofen lozenge compared with placebo [7-9]. Noteworthy was the demonstration of the onset of significant differentiation of flurbiprofen 8.75 mg from placebo at the first assessment time point (one hour) on all rating scales, in peak effects at one to three hours, and duration lasting for up to four hours on two of the three scales used to measure hourly effects over six hours. These effects represented at least a 20% reduction in the severity of each patient-reported outcome for the majority of flurbiprofen-treated patients: the pharmacologic effects of this low dose of flurbiprofen were distinctly differentiated from the demulcent effects of the lozenge base itself.

While this study did achieve its objective of demonstrating pharmacologic activity after multiple doses of study medication, there was one major limitation. Approximately 40% of the patients reported the onset of throat symptoms in the previous three days (and five patients were inadvertently admitted with onset in the previous four to five days). Therefore, the first 24 hours of the study was actually the fourth, fifth, or sixth day of these patients’ symptoms. Acute sore throat is an illness of short duration, with 85% improving within seven days [6]. This natural course may explain why the differences between active drug and placebo over days two to seven did not reach statistical significance in the trial. To improve assay sensitivity after the initial stages of sore throat (when symptoms are likely to be worst), future multiple-dose studies on sore throat treatments should restrict recruitment to patients with an onset of only one or two days.

## Conclusions

In this study, using specific design and methods, the sore throat (pharyngitis) pain model demonstrated that multiple doses of flurbiprofen 8.75 mg lozenges are an effective, well-tolerated treatment for patients with sore throat. Efficacy was evident for the patient-reported outcomes of pain intensity, pain relief, and swollen throat as well as throat function (swallowing) and overall patient satisfaction. We therefore conclude that this pain model is a practical and efficient multiple-dose assay for the safety and efficacy of analgesic agents.

## Abbreviations

AE: Adverse event; ANOVA: Analysis of variance; AUC: Area under the curve; BOCF: Baseline observation carried forward; CFR: Code of Federal Regulations; CI: Confidence interval; DSS: Difficulty Swallowing Scale; FDA: Food and Drug Administration; h: Hour; LS: Least square; PrAoI: Practitioner’s Assessment of Inflammation; SD: Standard deviation; SPID24: Summed pain intensity difference over 24 hours; STPIS: Sore Throat Pain Intensity Scale; STRRS: Sore Throat Relief Rating Scale; SwoTS: Swollen Throat Scale; TPA: Tonsillo-Pharyngitis Assessment; URTI: Upper respiratory tract infection.

## Competing interests

This study was funded by a research grant from Reckitt Benckiser Healthcare International Ltd., UK. SA, AS, TS, and GS are employees of Reckitt Benckiser Healthcare International Ltd. BS and ES received an investigational grant from Reckitt Benckiser Healthcare International Ltd., UK, to design and conduct this study.

## Authors’ contributions

All of the authors have contributed substantially to the study reported in this manuscript and the development of this manuscript: BS, SA, AS, and ES contributed to the study concept and design. GS performed the statistical analyses. BS, TS, and ES were involved in the conduct of the study and acquisition of data. BS, SA, AS, TS, GS, and ES contributed to the interpretation of results. All authors have reviewed and approved this manuscript.
